# FORENSIC NEUROFEEDBACK AND PREFRONTAL WORKOUT, A SURVEY

**DOI:** 10.1192/j.eurpsy.2023.1865

**Published:** 2023-07-19

**Authors:** I. S. Lancia, G. M. Festa, M. E. Candia, L. M. Colone

**Affiliations:** 1 Interdisciplinary Institute of Higher Clinical Education (IAFeC); 2 Pontifical Faculty of Educational Sciences «AUXILIUM», Rome, Italy

## Abstract

**Introduction:**

The logic behind the neuropsychological rehabilitation of the impulsive criminal is to treat the behavioral manifestations of these individuals as the product of a real pre-frontal syndrome, capable of causing deficits in the planning of behavior, in self-regulation, in the inhibition of impulsiveness and more generally in social and interpersonal skills. With the term prefrontal workout, literally “prefrontal workout” Eagleman (2011) refers to a real form of rehabilitation intended for subjects characterized by strong impulsive tendencies. Neurofeedback is used in prefrontal training. This technique mainly uses electroencephalography (EEG) and functional magnetic resonance imaging (fMRI) as indicators of brain function.

**Objectives:**

The premise of prefrontal training is that through rehabilitation, and therefore repeated practice, the frontal areas of our brain can be trained in order to improve the “control” of subcortical circuits and limbic areas responsible for impulsive and potentially destructive behavioral forces. Although this rehabilitation proposal does not specifically concern deviant individuals, it is intended for subjects with impulsive tendencies and difficulty in repressing a stimulus-seeking behavior, skills that fall within the category of frontal functions.

**Methods:**

It is possible that similar strategies can also be used effectively against deviant individuals, working to improve their ability to inhibit a behavioral tendency and reinforcing everything with real-time feedback. There is a very high incidence of attention deficit, hyperactivity disorder and related symptoms among people convicted of crimes, and a great many criminal acts involve impulsive behavior or loss of emotional control such as anger. Better control of behavior and emotions are among the most commonly reported outcomes of neurotherapeutic treatment.

**Results:**

Research and clinical experience also demonstrate the positive effects of neurofeedback with alcohol and drug abuse and depression, both common accompaniments of criminal behavior ( Fielenbach S. 2019; Margarita R. 2016; Konicar L. 2015).

**Conclusions:**

Certainly, research in this field is at a preliminary stage, and the limitations of these techniques are numerous. There are, for example, several doubts about which is the best strategy for the patient to use to control mental activity and inhibit, for example, the search for the substance to which one is addicted. Although promising, the literature highlights sometimes conflicting results: in this regard, see the meta-analysis by Cortese and colleagues (2016) and the document published by Youcha and colleagues (2008).

**Disclosure of Interest:**

None Declared

